# A Case of Acute Hepatitis E Infection in a Patient with Non-Hodgkin Lymphoma Treated Successfully with Ribavirin

**DOI:** 10.1155/2017/8941218

**Published:** 2017-01-15

**Authors:** Hasan N. Y. Haboubi, Rizwan Diyar, Ann Benton, Chin Lye Ch'ng

**Affiliations:** ^1^Department of Hepatology, Singleton Hospital, Sketty Lane, Swansea SA2 8QA, UK; ^2^Department of Haematology, Singleton Hospital, Sketty Lane, Swansea SA2 8QA, UK

## Abstract

We present the case of a man who, following immunosuppressive treatment for non-Hodgkin lymphoma, became infected with viral hepatitis E. Acute hepatitis E virus infection should be considered in patients with deranged liver function on a background of haematological malignancies or immunosuppression, even without travel to endemic regions. Whilst clearance is usually spontaneous in immune-competent individuals, these at-risk groups may develop a more complicated and protracted disease course. Thus awareness is important as additional treatment with ribavirin or pegylated interferon may be required, as in this case, in order to help achieve eradication.

## 1. Case Report

A 62-year-old man was found to have a spinal mass and was diagnosed with non-Hodgkins lymphoma in July 2014 and commenced R-CHOP (rituximab, cyclophosphamide, doxorubicin, vincristine, and prednisolone) chemotherapy in early August 2014. Following the 4th cycle of chemotherapy, abnormal liver function tests were discovered on routine bloods and he was subsequently referred to the hepatology clinic for investigation. The patient was asymptomatic and apart from lymphoma there was no significant past medical history. He was not on any regular medications.

He was nonsmoker and drank alcohol only occasionally. There was no history of travel to hepatitis endemic areas or relevant family history.

Clinical examination did not reveal any stigmata of chronic liver disease. Liver function tests showed raised Alanine Aminotransferase (ALT) of 707 (0–35 U/L), Aspartate Aminotransferase (AST) 341 (0–37 U/L), Alkaline Phosphatase (ALP) 161 (44–147 U/L), and bilirubin 7 (<22 *μ*mol/L). Synthetic function was normal. Screening for viral hepatitis A, hepatitis B, hepatitis C, HIV, and CMV was negative. Liver autoantibody screen was also normal. Ultrasound examination of liver was normal as well.

Further investigations detected hepatitis E RNA with a viral load of 5.9 × 10^6^ iU/mL and it was of genotype 3. A blood sample taken before chemotherapy was analysed (31/07/2014) for the presence of hepatitis E RNA, which returned as negative.

The presence of liver dysfunction without an immune response with seroconversion raised a suspicion of an alternative diagnosis. A liver biopsy was therefore performed. This demonstrated mild inflammation of the portal tracts with no ductopenia or granulomas present. There was moderate lobular lymphocytic infiltration with numerous apoptotic cells. No Mallory's hyaline, cholestasis, or liver cell dysplasia was seen. Special stains were negative for alpha-1-antitrypsin, HepBsAg, or copper-binding protein. PAS staining demonstrated abundant macrophages containing cell debris but there was no fibrosis. These findings were consistent with an acute lobular hepatitis, in keeping with acute hepatitis E infection.

**Figure 1:**
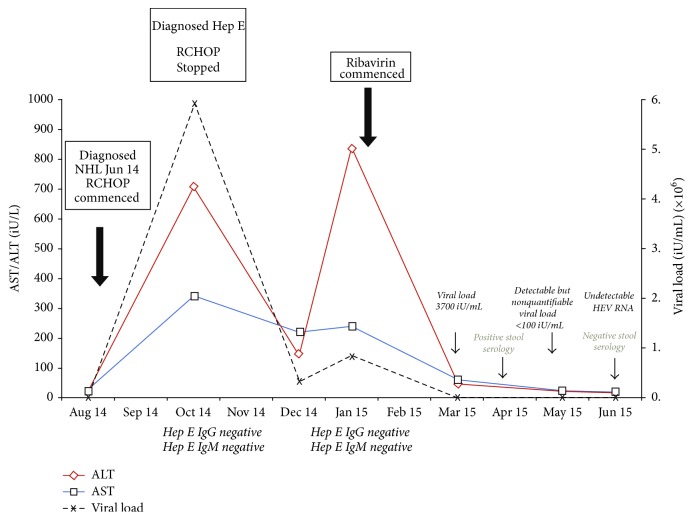
Timeline of laboratory tests demonstrating HEV viral load and derangement of liver function following commencement of R-CHOP chemotherapy. Following initial improvement with stopping of chemotherapy, worsening of liver function was observed. Full eradication and normalisation of liver enzymes were subsequently achieved following ribavirin treatment.

As the patient was asymptomatic he was initially managed by withholding consolidation R.CHOP chemotherapy and close monitoring of his liver function (October 2014, see Figure 1.). However after initial improvement in liver function (ALT reduced from 707 U/L to 141 U/L), the ALT started to rise again and peaked in late December 2014 to a level of 837 U/L. This coincided with an increase in viral load (RNA) from 3.3 × 10^6^ to 8.3 × 10^6^ iU/mL. Therefore it was decided to commence him on ribavirin treatment (500 mg twice daily) in January 2015. Viral load fell to 3700 iU/mL within one month of treatment (Figure 1). Liver function started to improve with return to normal within 2 months of treatment and no features of ribavirin induced anaemia were noted throughout treatment: haemoglobin at start of treatment was 139 g/L compared with 151 g/L at completion of treatment (reference ranged 135–162 g/L).

His hepatitis E RNA levels became undetectable in the blood after 4 months of therapy (April 2015) but stool serology was still positive and as viral shedding in the stool has been associated with viral rebound following cessation of therapy, he was continued on ribavirin 400 mg twice daily.

As it was deemed disadvantageous to recommence chemotherapy whilst still showing detectable evidence of hepatitis E activity, the patient was offered radiotherapy to his spine. In June 2015 (6 months after commencing ribavirin treatment) his stool serology was negative and viral load remained undetectable. Ribavirin was stopped and repeat imaging showed a reduction in the paravertebral mass, but disease was still present. He was thus recommenced on RCHOP chemotherapy and in August 2015 PET CT imaging showed evidence of complete remission. His liver function remains entirely normal.

## 2. Discussion

Hepatitis E virus (HEV) is an RNA virus with 4 genotypes. In endemic areas spread occurs by faecally contaminated water. In developed countries most cases are associated with travel to endemic areas; however cases of autochthonous HEV have been reported [1]. Genotype 3 virus usually causes these autochthonous cases in the developed world. Various animals including pig, deer, and rodents have been proposed as reservoir for HEV in nonendemic areas [2].

Acute hepatitis caused by HEV is usually self-limiting, though it has been shown to cause fulminant hepatitis in pregnant women [3]. Jaundice and loss of appetite is the most common presenting symptoms. Liver function shows elevated serum bilirubin, ALT, and AST, which could last for approximately 7 weeks [4]. Treatment for acute HEV infection is supportive. However discontinuing immunosuppressant medications and/or treatment with ribavirin may be required in some patients. This includes patients receiving methotrexate, antitumor necrosis factor alpha agents, rituximab, abatacept, tocilizumab, and corticosteroids [5].

HEV can evolve to chronic infection in immunosuppressed patients particularly those with solid organ transplant, hematological malignancies, and HIV positive patients with low CD4 count [6]. Reducing dose of immunosuppressive medications in posttransplant patients has been shown to help clear the virus. Monotherapy with ribavirin or pegylated interferon has been shown to be effective in treating chronic HEV in people with hematological disease or HIV [6]. There is no data on duration of treatment.
